# How to meet your ‘silent partner’: tips for approaching editors at conferences

**DOI:** 10.1186/s13059-019-1820-2

**Published:** 2019-09-17

**Authors:** Yixin Yao

**Affiliations:** Genome Biology, BMC, Shanghai, 200040 China

It is Monday morning, the end of your tedious department faculty meeting. As if the meeting has not been dreadful enough, your colleague starts to brag about how smooth their recently accepted submission has been and how it was solicited by several journals at the same time. You pat their back and wonder if you should ask them what they think that the journal editor had meant by ‘not broad enough interest’ in the last reject-without-review decision letter you received. The colleague had been put on tenure track at the same time as you, but everything seems curiously effortless for them, while you struggle to keep up. You are confident your work is no less significant than the accepted work that they are bragging about, and you can’t help but ask yourself whether you have missed something important.

Your mentors seem to talk to journal editors on a regular basis, and sometimes they even complain about too much correspondence. According to the rule of ‘six degrees of separation’, you are sure that you could get in touch with and eventually interest one of the editors with the details of your recently rejected work. You start planning which colleague you should reach out to first: your postdoc mentor, your PhD mentor or the ‘lucky’ colleague who you are sure will take the next open tenure position in your department.

You probably do not know it, but you have probably missed three to five editors at each of the conferences you attended last year, a total of at least ten conferences involving numerous cross-continental sleep-depriving flights. The journal editors sit in front of you while you give the talk and they stare at your poster while you explain the technical details of the experiments. They are observers that tend to be as silent as possible, but they aim to learn as much as they can. Their role as your career partner is written between the lines of an editor’s job description, yet getting to know or talk to a journal editor could seem daunting at first. Here are some tips for those seeking to meet a ‘silent partner’ at a conference.

## Conference book email addresses

Some conferences have ‘meet the editor’ panels, with the participants listed in the meeting program. You can go to the program website to check for these panels or lists. Some conferences are co-organized by journal editors, in which case their names will be listed on the front page of the website.

If you are having trouble finding this information on the conference website, once you are registered to a conference, you will be able to access the list of attendees, which might be on the printed version of a conference book or on a mobile application. The domain names of the attendees’ email addresses will be one of the most straightforward ways to identify professional in-house editors. On the other hand, it might take more effort to identify academic editors; you might need to cross-reference journal websites with the conference attendee list to find the editor whose journal interests you.

Schedule a 30-min meeting with the editor by email if you are not sure that you will be able to recognize them when you bump into them. Editors are generally very happy to meet with you and to answer any questions that you might have about a future or past submission. Another option would be to talk informally during a coffee break, but you should expect that you might only get a 5-min slot to do so. Although it may be very challenging to interest an editor in such a short time frame, you may still be able to hear comments from a completely new perspective.

People might argue that meeting in person is outdated; emails, phone calls or social media are efficient enough. This is a valid argument, but you might be the kind of person that finds a smile or some other form of body language helpful when interpreting the feedback regarding the ‘interest level’ of your work. The best editor might even give some brainstorming ideas, which could sound outrageous but which might help you to think outside of the box. In most cases, the editors will let you know how robust the findings need to be or how broadly applicable the work has to be for the journal to send it for review.

## Elevator talk: keywords

A good ‘elevator talk’ is a small talk that captures the audience’s attention within a few sentences. Aside from elevators, beware of editors when you grab your food, they will probably be waiting in the same line as you to grab a coffee or a sandwich lunch. Abstract thoughts are built, logged and archived by keywords and phrases; therefore, precise keywords are helpful to have on hand in order to capture the attention you need. A few good key thoughts will encourage an editor to come up with questions; these questions are indications of what’s going on in their minds and are those that are often asked in review reports circulating in this journal.

It’s always a good habit to prepare an ‘elevator talk’, which should evolve with the work in progress. When preparing your talk, try to contain one keyword that highlights the gap in the field that is filled by your work, one keyword to show the advances achieved by the work (which could be a new technique or a significant insight), and finally, one keyword to show which doors this work has opened for fellow scientists.

When you meet with an editor at the conference, if you don’t have time to set up a full meeting, you could mention that you have a talk or a poster scheduled at the conference. Invite the editor to your poster; they won’t miss the chance to hear an interesting story.

## Presentations: make yourself visible

You are center stage when you are chosen to give a 5–20 min talk. Remember that you are a storyteller, not a young PI who needs funding, good postdocs and published, well-cited articles. Don’t worry about the clicking sound of people taking pictures of you and your slides. Don’t worry about the questions that are going to bury you 5 min later. Tell a complete story first.

A complete story should describe the incentive behind your work, which shouldn’t be the fact that you happened to inherit some rare lab material or that your funding agency is favoring this topic lately. Go back to the keyword describing the gap knowledge that needed to be filled; this is the ‘once upon a time’ that you are looking for in a conference talk. The story should also contain a sound logic flow. You may be worried about sharing unpublished results as part of your talk, but leaving these results out of your slides can compromise your logic flow. In addition, editors are often most interested in your unpublished results and like to hear about where you think the project is heading.

Demonstrating a good understanding of the field and maintaining a sound logic flow throughout your talk is important in attracting your ‘silent partners’ because these are essential to making your project stand out among other scientific communications. It might not be immediately obvious, but peer review and eventually published articles are forms of scientific communications, and only good ones inspire followers. Mention the subject areas that you are interested in at the end of the talk; an editor may take the hint and come to catch up with you.

It might seem that posters have less impact than a talk at the moment of presentation, but posters are gold mines for editors because they often contain preliminary work with good potential, whereas many talks reference already published projects. As posters don’t have the luxury of verbal context or the length of a full manuscript, you should make sure that your poster effectively communicates your main results. The only visible text on posters might be the title and a few words on the figures, so make good use of them and make sure that they explain your work clearly.

Again, keywords come in handy when developing your title and making an impression. More time should be spent on the figures selected for the poster. They don’t have to be the same as those in the manuscript that you are writing, and they could be replotted to make a better illustration that is more suitable for a poster. In some cases, including all of the figures from your results might not be a good idea for a poster. How you plot the figures will be critical in helping the audience to understand what has been done and how significant your findings are. The figures should be self-explanatory without detailed legends.

## Conclusions

Scientific communication takes many forms, but conferences are places where direct communication with editors can happen in a quick and straightforward manner. Aside from promoting your work to the editors, don’t shy away from volunteering yourself as a reviewer, have your electronic resume prepared and offer to send it to the editor if they show interest. Your publication list can help the editor to see what your areas of expertise are. Prepare for different personal encounters, and you are sure to meet your next career partner very soon.

